# Annual Research Review: Umbrella synthesis of meta‐analyses on child maltreatment antecedents and interventions: differential susceptibility perspective on risk and resilience

**DOI:** 10.1111/jcpp.13147

**Published:** 2019-10-30

**Authors:** Marinus H. van IJzendoorn, Marian J. Bakermans‐Kranenburg, Barry Coughlan, Sophie Reijman

**Affiliations:** ^1^ Department of Psychology, Education, and Child Studies Erasmus University Rotterdam Rotterdam The Netherlands; ^2^ Department of Public Health and Primary Care School of Clinical Medicine University of Cambridge Cambridge UK; ^3^ Clinical Child and Family Studies Faculty of Behavioural and Movement Sciences Vrije Universiteit Amsterdam Amsterdam The Netherlands

**Keywords:** Child maltreatment, interventions, umbrella synthesis, meta‐analysis

## Abstract

Child maltreatment in the family context is a prevalent and pervasive phenomenon in many modern societies. The global perpetration of child abuse and neglect stands in stark contrast to its almost universal condemnation as exemplified in the United Nation’s Convention on the Rights of the Child. Much work has been devoted to the task of prevention, yet a grand synthesis of the literature is missing. Focusing on two core elements of prevention, that is, antecedents for maltreatment and the effectiveness of (preventative) interventions, we performed an umbrella review of meta‐analyses published between January 1, 2014, and December 17, 2018. Meta‐analyses were systematically collected, assessed, and integrated following a uniform approach to allow their comparison across domains. From this analysis of thousands of studies including almost 1.5 million participants, the following risk factors were derived: parental experience of maltreatment in his or her own childhood (*d* = .47), low socioeconomic status of the family (*d* = .34), dependent and aggressive parental personality (*d* = .45), intimate partner violence (*d* = .41), and higher baseline autonomic nervous system activity (*d* = .24). The effect size for autonomic stress reactivity was not significant (*d* = −.10). The umbrella review of interventions to prevent or reduce child maltreatment showed modest intervention effectiveness (*d* = .23 for interventions targeting child abuse potential or families with self‐reported maltreatment and *d* = .27 for officially reported child maltreatment cases). Despite numerous studies on child maltreatment, some large gaps in our knowledge of antecedents exist. Neurobiological antecedents should receive more research investment. Differential susceptibility theory may shed more light on questions aimed at breaking the intergenerational transmission of maltreatment and on the modest (preventive) intervention effects. In combination with family‐based interaction‐focused interventions, large‐scale socioeconomic experiments such as cash transfer trials and experiments with vouchers to move to a lower‐poverty area might be tested to prevent or reduce child maltreatment. Prevalence, antecedents, and preventive interventions of prenatal maltreatment deserve continuing scientific, clinical, and policy attention.

## Abstract 2

Read the Commentary on this article at doi: 10.1111/jcpp.13175

## Introduction

### What is child maltreatment?

Child maltreatment in the family context is a widespread phenomenon affecting the lives of millions of children all over the world. Broadly defined, child maltreatment refers to any interaction or lack of interaction reasonably within the control of a parent or person in a position of caregiving responsibility, that does (potential) harm to the child’s health or physical, mental, spiritual, moral, or social development in the context of the society in which the child grows up (see World Health Organization, 1999). Thousands of empirical studies on the prevalence, antecedents, and consequences of child maltreatment have been conducted, as well as hundreds of studies on (preventive) interventions of child maltreatment. These studies have been reviewed and combined quantitatively in a heterogeneous body of meta‐analyses for different types of maltreatment and with varying numbers of studies included. This hampers an overview of the field, in particular to balance the import of various antecedents of maltreatment and to make a considered decision about the antecedents that might be targeted best in preventive or curative interventions. As such, a grand synthesis of the literature that allows for comparing effect sizes of antecedents of child maltreatment and the effectiveness of interventions to prevent or reduce child maltreatment is required. Here we aim to provide such a synthesis using the umbrella synthesis approach (Ioannidis, 2009) to review and integrate the meta‐analyses of primary empirical studies on child maltreatment and to detect gaps in the literature.

The World Health Organization (WHO) describes the different types of child maltreatment in the Report of the Consultation on Child Abuse Prevention (1999). This report differentiates between the following types of maltreatment: child sexual abuse, physical abuse, emotional abuse, neglect including physical neglect, emotional neglect and educational neglect, and witnessing family violence which has recently been added to the catalogue of maltreatment types, and suggested to carry possibly serious developmental consequences (Van Rosmalen‐Nooijens et al., 2017). Most studies do not differentiate between the various types of child maltreatment, making it difficult to discover their discrete antecedents, correlates, and consequences. One of the reasons for not distinguishing between the various maltreatment types is the rather high degree of co‐occurrence, comorbidity, or poly‐victimization in child maltreatment: Adverse childhood experiences tend to cumulate and cluster within families (Finkelhor, Turner, Hamby, & Ormrod, 2011). In the current synthesis, we focus on the social, psychological, neurobiological, and intergenerational antecedents and correlates of child maltreatment and on the effectiveness of interventions to prevent or reduce such maltreatment.

The possible consequences of child maltreatment for development across the life span are beyond the scope of the current paper, and worth an umbrella synthesis in itself. In brief, meta‐analytic evidence shows that different types of child maltreatment are associated with a variety of mental and physical health problems across behavioral and biological developmental domains (e.g., Riem, Alink, Out, van IJzendoorn, & Bakermans‐Kranenburg, 2015; Trickett, Negriff, Ji, & Peckins, 2011). Although it is difficult to establish the causal direction between the experience of childhood maltreatment and later developmental issues, while excluding third factors leading to spurious associations, it is beyond reasonable doubt that maltreatment leaves its negative marks on lifelong development.

### How prevalent is child maltreatment?

The global prevalence of child maltreatment was estimated in a review of 244 studies with 551 prevalence rates covering *N* = 856,765 individuals (Stoltenborgh, Bakermans‐Kranenburg, Alink, & van IJzendoorn, 2015). The majority of the prevalence papers dealt with sexual abuse only (130 papers), and most prevalence studies were conducted in the USA. The overall worldwide estimated prevalence rates for self‐reported maltreatment studies (mainly assessing maltreatment ever experienced during childhood) were 127/1,000 for sexual abuse (76/1,000 among boys and 180/1,000 among girls), 226/1,000 for physical abuse, 363/1,000 for emotional abuse, 163/1,000 for physical neglect, and 184/1,000 for emotional neglect.

The prevalence rates were additionally estimated based on a best‐evidence synthesis (Prevoo, Stoltenborgh, Alink, Bakermans‐Kranenburg, & van IJzendoorn, 2017), that is, by selecting the highest quality studies with a priori methodology criteria (sampling procedure, sample size, response rate, and the use of a validated instrument [see (Slavin, 1995)]. Compared to the estimates from all available studies, the best‐evidence studies showed a lower estimate for sexual abuse (98/1,000) and a higher estimate for emotional neglect (261/1,000). For physical and emotional abuse, best‐evidence estimates did not differ significantly from the overall estimates.

An important issue in estimating the prevalence of child maltreatment is the vast difference in estimates between self‐reports and informant reports. Informant reports are mostly reports by professionals such as teachers alerting child protective services (CPS). The overall estimated prevalence rates for studies using informants were 4 per 1,000 for sexual abuse and 3 per 1,000 for both physical and emotional abuse (Stoltenborgh et al., 2015). These estimates are thirty to hundred times lower than those based on self‐report. Part of the explanation for the huge difference is that informant studies usually report about a limited period of time, ranging from a few months to a year, whereas most self‐report studies concern life‐time maltreatment experiences. Informants are likely to underreport, since they are only aware of the visible, small tip of the proverbial iceberg. Informant reports may thus underestimate prevalence rates, whereas self‐report studies might overestimate prevalence. Relatedly, the agreement between prospective and retrospective measures of childhood maltreatment has been shown to be poor (Baldwin, Reuben, Newbury, & Danese, 2019). With regard to comorbidity, few of the 244 prevalence studies assessed more than one type of maltreatment. Two studies reported the percentage of comorbidity, which was 35%–50% (Euser, Alink, Pannebakker, Vogels, Bakermans‐Kranenburg & van IJzendoorn, 2013; Scher, Forde, McQuaid, & Stein, 2004). Such high levels of comorbidity or poly‐victimization make it difficult to examine the unique antecedents of specific types of maltreatment and preclude an umbrella approach to this issue.

### What are risk and protective factors of child maltreatment?

From an evolutionary perspective, we might speculate about two explanations why child maltreatment has not been extinguished given its detrimental effects on maltreated offspring, which seem in conflict with evolutionary mechanisms (although maltreatment might be an example of a ‘spandrel’, that is, an accidental by‐product of adaptive selection; Gould & Lewontin, 1979). The first is the innate bias to promote one’s inclusive fitness which might be accompanied by abuse and even infanticide of infants that compete with procreation of one’s own offspring (Maestripieri & Carroll, 1998). For example, Hrdy (1977) observed infanticide in Langur primates by male dominant intruders who killed the newborns of females to accelerate procreation of their own offspring. Elevated levels of abuse in stepparent families would fit the picture emerging from this evolutionary background (van IJzendoorn, Euser, Prinzie, Juffer, & Bakermans‐Kranenburg, 2009). A second factor could be related to distribution of scarce resources to the infant with the best chances to survive, resulting in neglect of less fit, passive infants (‘angels’ going straight to heaven, Scheper‐Hughes, 1992). This selective preference for the most active and vocal infants might have played a role in the selective survival of Masai children with the more ‘difficult’ temperaments during the sub‐Saharan famine in 1974 (De Vries, 1984). In a similar vein, extreme stress caused by poverty and other dire straits might force parents to abandon some of their offspring to institutional care (van IJzendoorn, Palacios, Sonuga‐Barke et al., 2011). Many institutions with their regimented nature, low caregiver to children ratio, and high staff turnover deviate far from the Environment of Evolutionary Adaptedness (Bowlby, 1969) and consequently are highly detrimental to children’s development (van IJzendoorn et al., 2019).

In addition, parents who have been the victims of child maltreatment may be at heightened risk of maltreating their offspring. Studies on the intergenerational transmission of maltreatment are abundant, although equivocal in their findings. Sroufe, Egeland, Carlson, and Collins (2005) found that 70% of maltreated parents abused or neglected their own children, whereas Kaufman and Zigler (1987) in their study found that the majority of the parents with childhood maltreatment experiences did not maltreat their own offspring. More recent studies support the existence of intergenerational transmission of abuse, though transmission rates have varied according to whether studies used prospective or retrospective assessments of maltreatment and parent‐reported, child‐reported, or substantiated maltreatment. Transmission rates may also differ between maltreatment types. For example, a longitudinal, multi‐informant cohort study found that individuals who experienced maltreatment in childhood were more than twice as likely than their nonmaltreated counterparts to be reported to CPS as parents 30 years later. However, based on their children’s reports of maltreatment, intergenerational transmission was much weaker: Children of maltreated parents, compared with those of nonmaltreated parents, reported having experienced more sexual abuse on one out of three standardized retrospective measures used and reported more neglect (Widom, Czaja, & DuMont, 2015). There was no transmission effect on perpetration of physical abuse. Severity of experienced maltreatment may also influence the extent to which it is passed on to the next generation, with more severe maltreatment experiences increasing the likelihood of intergenerational transmission (Jaffee et al., 2013). These findings highlight the inherent complexity of the phenomenon and its measurement. Overall, high‐quality evidence seems to support the hypothesis that having experienced certain forms of (severe) maltreatment increases the chances of maltreating one’s own offspring, with a moderate to strong effect size (Schofield, Lee, & Merrick, 2013).

In addition to experienced child maltreatment, parents’ experience of interpersonal or intimate partner violence (IPV) and marital conflict, which is estimated to affect at least a quarter of young adults in the USA (Black et al., 2011), may by extension also harm their interaction with the child. The spillover hypothesis submits that marital quality is related to parent–child relationship quality (Engfer, 1988), and IPV may be particularly predictive of child abuse (Krishnakumar & Buehler, 2000), both because of the stress experienced by parents who are victim of IPV and because violence is modeled as a way to deal with interpersonal conflicts that may also emerge in the parent–child relationship.

Psychological and neurodevelopmental factors may also constitute risk factors for maltreatment, both in the parent and in the child. Parental characteristics (e.g., low threshold for aggression or low inhibitory control) and psychopathology (e.g., major depressive disorder or borderline personality disorder) may interfere with normative caretaking of young children (e.g., see Belsky, 1993). Parental psychopathology may be differentially related to type of maltreatment, with depressive symptomatology showing associations with parental neglect (Dubowitz, 1999) and anger and hyper‐reactivity associated with physical abuse (Stith et al., 2009). Regarding child characteristics, early studies (e.g., Belsky, 1993) tended to report associations between child factors and maltreatment although evidence remained equivocal. More recently, however, there is an emerging body of evidence that seems to indicate that children with certain neurodevelopmental features such as the presence of an autism spectrum disorder and/or learning difficulty may be at an increased risk of maltreatment (e.g., Dion, Paquette, Tremblay, Collin‐Vézina, & Chabot, 2018; McDonnell et al., 2019).

Potentially related to some of the abovementioned antecedents, parental neurobiological factors might play a role in increasing the risk for child maltreatment. A long‐standing idea is that the neurophysiology of perpetrating parents is dysregulated, which would make them constitutionally more liable to become abusive. Neural reactivity (Rilling & Young, 2014), as well as autonomic and sympathetic nervous system responses, prepares the individual organism for action to cope with stress, but if the balance between the sympathetic and parasympathetic systems is dysregulated, over‐ or underreactivity to stressful stimuli such as infant crying might result in harsh parenting or neglect (Reijman et al., 2016).

As context factors, single parenthood, lack of social support, and low socioeconomic status have been associated with increased risk for child maltreatment (Stith et al., 2009). We have previously shown that low income is related to lower parental sensitivity (Bakermans‐Kranenburg, van IJzendoorn, & Kroonenberg, 2005) and more insecure‐disorganized infant–parent attachment (Cyr, Euser, Bakermans–Kranenburg, & van IJzendoorn, 2010). The burden of stresses accompanying poverty may increase the risk for child abuse and neglect.

Whereas risk factors elevate the chance of maltreatment occurring in the child’s life, protective factors decrease these chances even when risk factors are present. In doing so, protective factors act as moderators of the association between risk factors and maltreatment. For example, the risk for maltreatment in families living in poverty – a risk factor for physical neglect – may be reduced if and when a social support network or intervention is in place. Protective factors might be situated at the individual (constitutional) level or at the level of the social context. At the individual level, for example, a parent may remain unruffled by the stressful impact of daily hassles, or a child may not trigger maltreatment on the part of his/her stressed parents because of his/her easy‐going temperament. Protection at the level of the social context may buffer the influence of parental or child risk factors, and longitudinal studies have provided evidence that the intergenerational cycle can be broken, for example, by the presence of safe, stable, and nurturing relationships with intimate partners and high levels of maternal warmth toward children (Jaffee et al., 2013; Merrick, Leeb & Lee, 2013; Schofield et al., 2013). It might be hypothesized that the interplay of risk and protective factors changes across development (Masten & Cicchetti, 2016).

**Figure 1:**
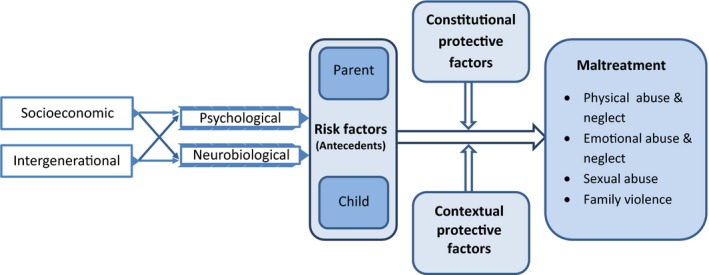
Risk and protective factors for child maltreatment. Note: Heuristic model of risk and protective factors for child maltreatment, with socioeconomic adversities and intergenerational experiences as more distal factors that are suggested to influence type and chronicity of maltreatment, mediated by more proximal factors on the psychological and neurobiological level. Distal and proximal influences are moderated by parent or child protective factors (constitutional characteristics, e.g., easy temperament) and protective factors in the social context (e.g., parent coaching intervention) as predicted by differential susceptibility theory. [Colour figure can be viewed at http://www.wileyonlinelibrary.com]

In Figure 1, a model of risk and protective factors for the occurrence of child maltreatment is presented. This is a heuristic model that guides our synthesis as it draws attention to the various proximal and distal levels of risks that have been more or less systematically addressed in maltreatment research. Most research on antecedents of child maltreatment has focused on risk factors, and not much attention has been paid to protective factors in the parent, child, or wider social environment. We consider intervention studies to prevent or reduce child maltreatment as aiming at bolstering potentially protective factors. It follows that successful interventions might also reveal the constitutional or contextual buffers against the risks for maltreatment. The model of risk and protective factors for child maltreatment might also point to gaps in our knowledge that need to be addressed in future work.

### Is it possible to prevent or reduce child maltreatment?

In view of the high prevalence of maltreatment and the negative consequences for child developmental outcomes, effective intervention programs to prevent and reduce maltreatment are crucial, and indeed, the number of parent support programs has steeply increased over the last decades. Parents in stressful or low‐resource conditions are entitled to be supported in their struggle to respond appropriately to challenging child behavior, and child maltreatment interventions may benefit from incorporating parent training that enables them to set limits to challenging child behaviors in less intrusive and more sensitive ways (Moss, Dubois‐Comtois, Cyr, Bernier, & St‐Laurent, 2017; Juffer, Bakermans‐Kranenburg & van IJzendoorn, 2017).

Preventive interventions focus on child abuse potential. They vary from informing parents in the general population about effective parenting to programs targeting at‐risk families. The Nurse‐Family Partnership is an example of the latter type of programs, with a focus on pregnant adolescent low‐income women, often single and without social support (Olds, Henderson, Chamberlin, & Tatelbaum, 1986). Through nurse home visits prenatally and during the first two years of the child's life, mothers’ health behavior during and after pregnancy is promoted, mothers are stimulated to establish supportive relationships, and they are linked to other services as needed. For families with documented abuse or neglect, programs that aim at reducing maltreatment have been developed. Parent–child interaction therapy (PCIT, Chaffin et al., 2004; Eyberg & Robinson, 1982) is an example of a targeted program that aims to reduce the incidence of child maltreatment in physically abusive parents. The intervention consists of child‐directed interaction training in 14 weekly one‐hour live‐coached sessions, in which the parent is instructed to follow the child's lead, but is also taught to direct the child's behavior and use consistent disciplinary techniques.

A number of meta‐analyses on the effectiveness of intervention programs aimed at reducing child abuse potential or child maltreatment have been conducted, some including only randomized controlled trials, others a mixture of both randomized and nonrandomized studies. In our umbrella synthesis of 16 meta‐analyses of pertinent maltreatment intervention studies (see below), we focused on the best evidence available in this complicated but crucial area of research, derived from randomized studies.

## The current study: An umbrella synthesis of meta‐analyses

Over the years, hundreds of empirical studies on the antecedents of child maltreatment and on (preventive) interventions of child maltreatment have been conducted. It has become difficult to get an overview of the rapidly expanding empirical literature and to distill the best evidence from the highly heterogeneous work. Even the number of meta‐analyses has increased rapidly in the past decade, and it seems timely to take stock of the available evidence. We use the so‐called umbrella synthesis approach (Ioannidis, 2009) to analyze and integrate the meta‐analyses of primary empirical studies. An umbrella synthesis involves the systematic collection and assessment of multiple meta‐analyses published on a specific topic: Meta‐analytic results are integrated and reviewed following a uniform approach to allow their comparison. The umbrella approach leads to comparable estimates of combined meta‐analytic effect sizes, an evaluation of the heterogeneity and potential biases, a systematic stratification of the evidence, and, if possible, sensitivity analyses to identify potential reporting biases (Fusar‐Poli & Radua, 2018). The quality of published meta‐analyses is systematically coded, taking into account the search strategy, numbers of studies included, coding procedures, and tests for publication bias. This umbrella approach is meant to create an optimal evidence base for further theoretical and empirical studies, clinical practice, and policy in the area of child maltreatment, with a focus on antecedents of maltreatment and (preventive) interventions reducing (the risk of) child maltreatment, and to detect gaps in the literature to be closed in future research.

## Method

### Search strategy

The following electronic databases were searched to identify relevant meta‐analyses: Web of Knowledge (including the Web of Science Core Collection, Current Contents Connect, KCI‐Korean Journal Database, Medline, the Russian Science Citation Index, and SciELO Citation Index), Cochrane Library, and PubMed. A list of search terms for use in Web of Knowledge, Cochrane, and PubMed is presented in Table S1 in the Supporting Information. The search strategy used a three‐level approach and combined the population of interest (i.e., child and adolescents), the moderator/outcome of interest (i.e., child maltreatment), and the studies of interest (i.e., meta‐analytic reviews or quantitative syntheses). The search period covered studies published during the 5‐year period between January 1, 2014, and December 17, 2018. This period was chosen to include recent meta‐analyses most of which cover several decades of maltreatment research and to avoid too many overlapping studies included in the older meta‐analyses. Publications were identified by title and abstract search.

### Eligibility criteria

**Figure 2:**
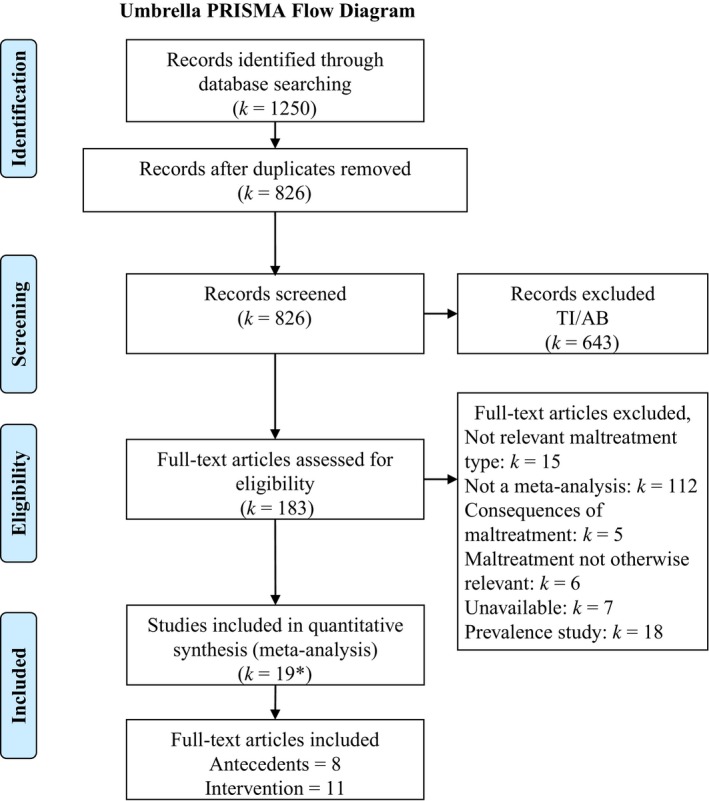
PRISMA flowchart of included and excluded studies. *Corrections to included papers (*k* = 1); TI/AB = Title/Abstract screening. [Colour figure can be viewed at http://www.wileyonlinelibrary.com]

The inclusion and exclusion criteria are presented in Table S2. Meta‐analytic reviews of studies that either reported on the antecedents or (preventive) interventions of maltreatment were included. We adopted a stepwise approach to screening papers in which we asked the following questions: (a) Is the study a meta‐analytic review? (b) Does the review contain information about maltreatment? (c) Is this information about intrafamilial child maltreatment? iv) Is there information about moderators/determinants of maltreatment/ interventions for preventing maltreatment? Intercoder agreement between two of the authors (BC and MHvIJ) for the screening phase was *kappa* = .85 (*k* = 30). For the PRISMA flow diagram of screening and selection of studies, see Figure 2.

### Quality rating

Meta‐analyses might differ with respect to the methods of the literature search, the assessment of the quality of the studies included, the extraction and statistical analysis of the study results, and the report of the meta‐analytic findings. For our umbrella synthesis of meta‐analyses on child maltreatment, we developed a coding system taking these various steps of the meta‐analytic process into account. The following features of the meta‐analyses were rated: the use of a systematic search strategy involving the PRISMA approach (Moher, Liberati, Tetzlaff, Altman, & The PRISMA Group, 2009); the number of studies included (with a minimum of *k* = 4 study outcomes); the number of participants included; type of study designs (cross‐sectional, [quasi‐] experimental; longitudinal; mixed); reports of homogeneity tests and moderator analysis; examination of publication bias and correction of the combined effect sizes if needed; study quality rating; and reported intercoder agreement for the various ratings of the studies (see Figure S1 for the coding form). Intercoder agreement between two authors (BC and MJBK) for the various quantitative features (e.g., number of studies and subjects) and effect size extraction was 90% exact agreement (*k* = 13). Intercoder agreement for the quality ratings (e.g., systematic search, publication bias analysis) was 74% exact agreement (*k* = 13).

Overall quality rating on a scale from (1) very low to (5) very high was based on whether or not the literature search was done in a systematic way (e.g., PRISMA guidelines); publication bias was examined, and trim‐and‐fill or other ways of dealing with publication bias were applied; homogeneity was tested; quality ratings of individual studies were reported; and whether intercoder agreement was reported for literature search, for coding of study characteristics, for quality ratings of individual studies, and for the extraction of effect sizes. Intercoder agreement for the overall quality rating of the meta‐analyses (intraclass correlation, single measure, exact agreement) was ICC = .79.

### Data extraction and harmonization

In several papers, more than one potentially relevant ES was presented, for example, for various types of child maltreatment. Where possible, effect sizes for each of the maltreatment types were extracted. The same goes for subgroups of studies distinguished by moderators such as gender: combined effect sizes for each of the groups of studies were separately coded. The effect size of the largest study in a meta‐analysis was also extracted to present the most precise estimate with the smallest confidence interval. When two or more reviews included a complete or substantial overlap in primary studies, the most recent or broader meta‐analysis was used. Because in several meta‐analyses the effect sizes of the primary studies were not reported, we analyzed the data on the level of the meta‐analytic results. Combinations of meta‐analytic outcomes were estimated by adding combined effect sizes after transformation into Fisher z. We reported effect sizes in Cohen’s *d* to facilitate comparison across studies, meta‐analyses, and domains. The meta‐analytic effect sizes were transformed into Cohen’s *d* using the program Comprehensive Meta‐Analysis version 3.0 (CMA, Borenstein, Rothstein, & Cohen, 2005). Transformation of relative risks (RR) and odds ratios was done using https://www.psychometrica.de/effect_size.html#transform (Lenhard & Lenhard, 2016). In some cases, the transformations were approximations of the original effect size, for example, when information on number of subjects, prevalence of base‐rate risk, or the confidence interval around the point estimate was missing.

## Results

### Antecedents of child maltreatment

Eleven meta‐analyses (reported in eight papers) on the antecedents of child maltreatment were identified (see Table 1). Three of these included only studies on documented maltreatment, one focused on child abuse potential or self‐reported maltreatment, and the other seven meta‐analyses included studies with officially reported child maltreatment as well as studies on child abuse potential and self‐reported maltreatment. The numbers of studies included in the meta‐analyses varied from six studies on the link between intimate partner violence victimization and self‐reported emotional abusive parenting (Chiesa et al., 2018) to 84 studies on parental experiences of childhood maltreatment as predictor of maltreatment of offspring (Assink et al., 2018). The total sample sizes varied from somewhat <500 to over 8,500, but was not always reported. Effect sizes ranged between *d* = −.10 (for parental autonomic nervous system reactivity, Reijman et al., 2016) and *d* = .69 (for parental insecure attachment, Lo et al., 2017), indicating a broad range of meta‐analytic effects.

**Table 1:** Meta‐analyses of studies on antecedents of child maltreatment

Study	Pub year	Maltreatment	Antecedent	Design	*k*	*n*	Cohen’s *d*	95% CI	Homogeneity	Pub bias	ES trim	*N* largest study	ES largest study	Quality rating^a^
Assink et al.	2018	CM, SR	Parent’s CM	Mixed	84	>>1,000	.60	0.52, 0.69	1	3, *k* = 18 imputed	0.70	n.r.	n.r.	5
Chiesa et al.	2018	CM, SR EA	IPV	Mixed	6	5,798	.47	n.a.	1	0	n.r.	2,508	0.20	3
Chiesa et al.	2018	CM, SR PA	IPV	Mixed	15	8,637	.35	n.a.	1	1	n.r.	2,508	0.10	3
Kane et al.	2018	CM	Dependency of perpetrators	Mixed	21	1,321	.36	n.a.	0	0	n.r.	472	0.31	1
Lo et al.	2017	CM	Insecurity	Cross	10	1,090	.51	n.a.	2	2	n.a.	213	0.41	3
Lo et al.	2017	CAP	Insecurity	Cross	7	740	.69	n.a.	2	2	n.a.	276	0.67	2
Madigan et al.	2019	CM, SR	Parent’s CM	Mixed	80	>>1,000	.45	0.37, 0.54	1	2	n.a.	n.r.	n.r.	4
Mulder et al.	2018	CM, SR N	Low SES	Mixed	28	>>1,000	.34	0.13, 0.54	2	3, *k* = 5 imputed	0.48	n.r.	n.r.	5
Reijman et al.	2016	CM, CAP	HR baseline	Cross	10	492	.24	0.03, 0.45	2	0	n.r.	104	0.50	4
Reijman et al.	2016	CM, CAP	ANS reactivity	Cross	10	471	−.10	−0.36, 0.16	1	0	n.r.	83	0.00	4
Seto et al.	2015	CM, SA	Parent’s CM	Mixed	8	912	.31	0.15, 0.47	1	2	n.a.	n.r.	n.r.	4

n.a., not applicable; n.r., not reported; CM, child maltreatment (officially reported); CAP, child abuse potential; SR, self‐reported maltreatment; EA, emotional abuse; PA, physical abuse; N, neglect; IPV, interpersonal violence; SA, sexual abuse; cross, cross‐sectional.

Quality indicators: 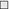 high; 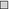 medium; 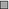 low.

^a^Quality rating: overall quality score of the meta‐analysis, including the reporting of intercoder reliabilities for search, moderator coding, and data extraction; higher scores represent higher quality.

**Figure 3:**
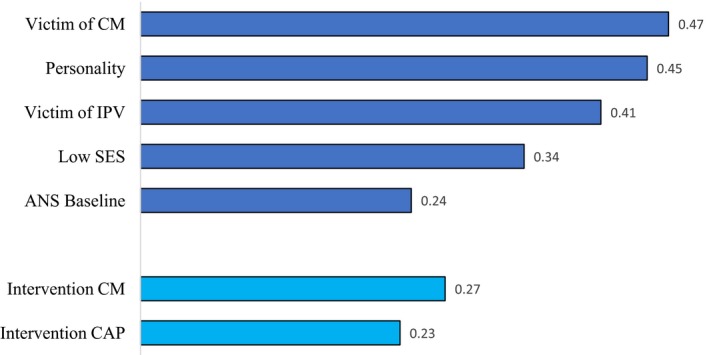
Umbrella effect sizes (*d*) for antecedents of child maltreatment and effectiveness of interventions to reduce or prevent maltreatment. Note: In this Figure, the effect sizes (Cohen’s d) combined across several meta‐analyses are presented, starting with parents having been victim of maltreatment in their own childhood, parental personality influences, having been victim of interpersonal or intimate partner violence (IPV), low socioeconomic status (SES), and baseline regulation of the autonomic nervous system (ANS). The final two antecedents are the results of interventions focusing on child maltreatment itself, and interventions targeting individuals with elevated risk of becoming a perpetrator (‘child abuse potential’ or CAP). All combined effect sizes are significant and in the expected direction. CM = Child maltreatment; IPV = Intimate partner violence; SES = Socioeconomic status; ANS = Autonomic nervous system; CAP = Child abuse potential. The effect size for ANS reactivity was *d* = −.10 and nonsignificant. [Colour figure can be viewed at http://www.wileyonlinelibrary.com]

In this set of meta‐analyses, we distinguished five categories of antecedents of child maltreatment: (a) parental experience of childhood maltreatment, representing intergenerational transmission of maltreatment, (b) parental experience of intimate partner violence (IPV), (c) parental personality characteristics, (d) parental baseline physiology and physiological reactivity, and (e) socioeconomic status (SES). The meta‐analyses in each of these categories are presented below, and overall umbrella effect sizes for the categories were computed (see Figure 3).

### Parental experience of childhood maltreatment

Several meta‐analyses examined transmission of experienced child maltreatment to perpetrating maltreatment in the next generation, and combining the meta‐analytic effect sizes in an umbrella effect size, this is the antecedent with the strongest link to certified or self‐reported child maltreatment (*d* = .47), see Figure 3. For example, in a meta‐analysis of 78 studies with 17,178 participants Seto et al. (2015) compared intra‐ versus extrafamilial offenders of child sexual abuse and found that intrafamilial offenders were more likely to have experienced sexual abuse themselves (*d* = .10; *k* = 20) or, more generally, to have experienced abuse or neglect in their family of origin (*d* = .31; *k* = 8). In another meta‐analysis on the intergenerational transmission of child maltreatment, significant transmission of maltreatment was found (*d* = .45; *k* = 80), with more specific transmission of type of abuse in case of emotional and sexual abuse and more diffuse transmission in case of physical abuse and neglect (Madigan et al., 2019).

The most comprehensive and best‐evidence meta‐analysis was conducted by Assink et al. (2018) on *k* = 84 studies, examining the transmission of physical, sexual, and emotional abuse and neglect. About half of the studies overlapped with the meta‐analysis of Madigan et al. (2019). The combined effect size amounted to *d* = .60, and after correction for publication bias, the combined effect size increased to *d* = .70. Because lower quality of studies was rather strongly associated with higher effect sizes, a set of high‐quality studies (*k* = 21) was selected to compute a best‐evidence combined effect size within this meta‐analysis, and these high‐quality studies provided a combined effect size of *d* = .49. Analyzing types of maltreatment separately, a lower effect of intergenerational transmission of child maltreatment was found for children who experienced physical abuse (mean *d* = .51) and neglect (mean *d* = .61) than for unspecified maltreatment types (mean *d* = .71) but similar differences were not found for the maltreatment types experienced by parents (Assink et al., 2018). Whether or not child maltreatment was officially recorded in the primary studies made a difference: Documented maltreatment of offspring led to a larger combined effect size of *d* = .76 compared with *d* = .47 in the other studies. Official recording of maltreatment experienced by the parents did not make a difference (Assink et al., 2018).

### Parental experience of intimate partner violence

IPV victimization as an antecedent of emotional abuse and physical abuse of children was meta‐analytically examined by Chiesa et al. (2018). The meta‐analyses included relatively modest numbers of studies (six for emotional abuse and fifteen for physical abuse), but total sample sizes were substantial (more than 5,500 and more than 8,500, respectively). The umbrella effect size for the association between IPV and child maltreatment was *d* = .41, quite similar to the strength of the association between experienced intrafamilial child maltreatment (see Figure 3). The largest study included in the Chiesa et al. (2018) meta‐analyses reported on 2,508 mothers from the Fragile Families and Child Well‐Being Study, 40% of whom had experienced IPV by their current partner. The effects in this largest primary study were substantially smaller (*d* = .20 for emotional abuse and *d* = .10 for physical abuse) than the meta‐analytic effect sizes for emotional and physical abuse (*d* = .47 for emotional abuse and *d* = .35 for physical abuse).

### Parental personality

Insecure attachment of the parents to their own parents has been speculated to predict elevated levels of child abuse potential as well as rates of officially recorded child maltreatment cases. In a meta‐analysis of 10 cross‐sectional studies (*N* = 1,090) on officially recorded maltreatment, Lo et al. (2017) found a 2.5 times elevated risk of child maltreatment in insecurely attached parents (*d* = .51), which was supported by the results of the largest study in the dataset (*N* = 213; Zuravin et al., 1996) showing a more than twice as high risk of child maltreatment in insecure compared with secure parents (*d* = .41). Mothers in this study were single and came from low‐income backgrounds with a majority having experienced abuse before the age of 18 years. Attachment to the mothers’ own primary caretaker was assessed with the self‐report Michigan Profile of Parenting (Zuravin et al., 1996). The combined effect size for *k* = 7 studies on child abuse potential amounted to a 3.5 times elevated risk (or *d* = .69) of child maltreatment in insecurely attached caregivers, compared to those with secure attachments (Lo et al., 2017). Interpersonal dependency is a personality trait that often accompanies insecurity and preoccupation with relationships, but is also related to aggression when a valued relationship is endangered or breaking down (Bornstein, 2006). The meta‐analysis of *k* = 21 studies (*N* = 1,321) on dependency and reported maltreatment (Kane & Bornstein, 2018) showed a combined effect size of *d* = .36. The umbrella effect size for the association between parental personality and child maltreatment was *d* = .45.

### Parental baseline physiology and physiological reactivity

Parental physiology was examined in two meta‐analyses, both with relatively modest total sample sizes, which is not surprising given the complicated measurements. Higher baseline heart rate was associated with increased risk for child abuse in a meta‐analysis of 10 studies with *N* = 492 participants. The combined effect size was significant but modest, *d* = .24. The largest study (*N* = 104) showed an effect size of *d* = .50 in parents of children with substantiated child maltreatment experiences (Creaven et al., 2014). The second meta‐analysis found no support for the hyper‐reactivity hypothesis, as reactivity of the autonomic nervous system to stress was not elevated in participants at risk for maltreatment (*d* = −.10).

### Low SES

On the level of environmental risk, socioeconomic status was a predictor of elevated risk for child maltreatment (*d* = .34). In a meta‐analysis of *k* = 28 studies on (CPS‐reported and self‐reported) neglect, Mulder et al. (2018) found a combined effect size of *d* = .34 (95% CI 0.13, 0.54) for low SES, which after trim‐and‐fill correction increased to *d* = .48 (95% CI 0.25, 0.71) (see Table 1). As an example of one of the primary studies included in this meta‐analysis, a nationwide maltreatment prevalence study in the Netherlands based on sentinel reports, CPS reports, and self‐reports showed that children from families with a low educational level, single‐parent families, immigrant families, and children with unemployed parents had a significantly increased risk to become a victim of child maltreatment (Euser et al., 2013), see Figure S2.

## Interventions to prevent or reduce child maltreatment risks

We found five meta‐analyses of interventions targeting child abuse potential or with self‐reported child maltreatment as outcome measures (see Table 2). Combined effect sizes for interventions focusing on a broad spectrum of child maltreatment risks (including child abuse potential and parental risk factors for child abuse) ranged from *d* = .13 (Euser et al., 2015) to *d* = .31 (Kennedy et al., 2016). The estimated overall effect size for child abuse potential and self‐reported child maltreatment was *d* = .23, see Figure 3. A best‐evidence meta‐analysis was presented in Euser et al. (2015) on the largest number of RCT studies (*k* = 27) with 4,883 families involved (see Table 2). This meta‐analysis found a small effect of parenting programs that disappeared after correction for potential publication bias using the trim‐and‐fill approach. Effective interventions appeared to be parenting programs involving parent training (*d* = .37; 95% CI 0.15–0.59), whereas interventions providing only support to the families did not succeed in bringing down the (risk of) maltreatment. Parenting seems to be a more powerful causal factor in child maltreatment than distal factors such as the wider social support context. Promoting healthy behaviors during pregnancy and early parenthood (Brayden et al., 1993), establishing social support networks (Bugental et al., 2002), or screening for developmental delay (Duggan et al., 2007) was not found to be effective in preventing child maltreatment. In contrast, significant effects were found for intervention programs offering parent training, such as Multisystemic Therapy for Child Abuse and Neglect (Swenson et al., 2010) and parent–child interaction therapy (PCIT; Chaffin et al., 2004), although in a more recent meta‐analysis of five PCIT studies (Kennedy et al., 2016, see Table 2) the combined effect size of *d* = .31 was not significant (95% CI ‐ 0.00–0.62). The probably most widely known and impressive long‐term broadband intervention with nurse home visitations (Olds et al., 1986) provided to a sample at high risk for maltreatment did show a slight, nonsignificant reduction in reports of child maltreatment to the child protective services within two years of the final sessions (12 reports in the intervention group, *n* = 181, and 16 reports in the control group, *n* = 161, see Viswanathan et al., 2018). Notably, this intensive, broadband nurse home visiting program showed impressive effects in other domains such as health and cognitive development.

**Table 2:** Meta‐analyses of intervention studies to prevent or reduce child maltreatment

Study	Pub year	Maltreatment	Intervention type	RCT	*k* studies	*N* meta	ES (*d*)	95% CI	Homogeneity	Pub bias	ES trim	*N* largest study	ES largest study	Quality rating^a^
Chen et al.	2016	CM	Parenting programs	RCT	8	>1,000	.21	0.02, 0.39	1	1	n.r	1,173	0.07	3
Chen et al.	2016	SR, CAP	Parenting programs	RCT	24	>1,000	.20	0.10, 0.30	1	1	n.r.	1,173	0.07	3
Dijkstra et al.	2016	CM	Family group conferencing	Mixed	18	1,334	.10		1	3	0.14	680	n.r.	5
Euser et al.	2015	CM	Parenting programs	RCT	6	423	.35	0.17, 0.53	1	0	n.r.	160	0.10	3
Euser et al.	2015	SR	Parenting programs	RCT	27	4,883	.13	0.05, 0.21	1	3, *k* = 9 imputed	0.02	992	n.r.	5
Hackett et al.	2016	CM	Domestic violence victim interventions	Mixed	5	>1,000	.55	0.42 ,0.69	2	0	n.r.	384	0.61	1
Kennedy et al.	2016	CAP	PCIT	Mixed	5	<1,000	.31	−0.00, 0.62	1	0	n.r.	151	0.20	2
O’Connor et al.	2018	CM prenatal	Alcohol abstinence	n.r.	5	796	.45	0.20, 0.70	2	0	n.r.	255	0.93	2
Park et al.	2017	CM	Home‐based programs	RCT	5	<1,000	.48	0.20, 0.76	0	2	n.a.	n.r.	n.r.	2
Park et al.	2017	SR, CAP	Home‐based programs	RCT	15	>1,000	.30	0.17, 0.43	0	2	n.a.	n.r.	n.r.	2
Van der Put et al.	2018	CM	Mixed	Mixed^b^	41	>>1,000	.21	0.12, 0.31	1	0	n.r.	n.r.	n.r.	3
Van der Put et al.	2018	CAP	Mixed	Mixed	62	>>1,000	.30	0.22, 0.37	1	0	n.r.	n.r.	n.r.	3
Viswanathan et al.	2018	CM removal	Home visiting	RCT	4	609	−.05	−1.10, 1.01	1	2	n.a.	263	0.82	2
Viswanathan et al.	2018	CM	Home visiting	RCT	10	2,434	.03	−0.11, 0.18	2	2	n.a.	1,060	0.17	5
Vlahovicova et al.	2017	CM (PA)	Parenting programs	RCT	4	<1,000	.15	−0.04, 0.34	2	0	n.r.	n.r.	0.14	2
Zhang et al.	2018	CM	Drug abuse interventions	Mixed	16	7,085	.31	0.18, 0.44	0	2	n.a.	1,220	0.69	2

n.a., not applicable; n.r., not reported; CM, child maltreatment (officially reported); CAP, child abuse potential or self‐reported maltreatment; PA, physical abuse.

Quality indicators: 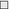 high; 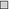 medium; 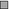 low

^a^Quality rating: overall quality score of the meta‐analysis, including the reporting of intercoder reliabilities for search, moderator coding, and data extraction; higher scores represent higher quality.

^b^Explains that the gray shading is linked to quality rating of the papers and how the quality score was determined.

Eleven meta‐analyses on interventions targeting substantiated child maltreatment cases were identified (see Table 2). Combined effect sizes for interventions focusing on reported maltreatment ranged from *d* = −.05 (Viswanathan et al., 2018) to *d* = .55 (Hackett et al., 2016), with an estimated umbrella effect size of *d* = .27 (see Figure 3). The best meta‐analytic evidence for interventions focusing on child maltreatment reported to child protective services may be derived from Viswanathan et al. (2018) who included ten RCT studies in their estimate of the effectiveness of interventions to decrease maltreatment (see Table 2). The combined effect size was nonsignificant, *d* = .03. Interventions provided in or referable from primary care did not consistently prevent child maltreatment in the short run (within less than a year), nor in the long run (after two years). No statistically significant associations were observed between interventions and outcomes for emergency department visits, hospitalizations, or prevention of death.

The largest effect size was detected in the meta‐analysis on prenatal interventions to reduce alcohol consumption during pregnancy (O’Connor et al., 2018). This RCT used a one‐session alcohol counseling intervention that showed a substantial impact with an effect size of *d* = .93 in a sample of *N* = 255 ethnically diverse, low‐educated pregnant women living in poverty, with a comprehensive baseline assessment of alcohol use at 18‐week gestational age and a post‐test at 36‐week gestational age (O'Connor & Whaley, 2007). In an individual face‐to‐face session, nutritionists provided participants with a standardized workbook consisting of education and feedback, cognitive behavioral procedures, tips for goal setting, and contracting. Pregnant women were instructed to stop drinking during pregnancy, and they in fact did reduce alcohol consumption drastically compared with the control group. It should be noted that alcohol consumption during pregnancy is not restricted to low SES families; on the contrary, some studies show that the habit of drinking wine on a (almost) daily basis is customary in middle and high SES families and in more than one‐third of the families tends to continue during pregnancy (Bakker et al., 2010). As alcohol may have detrimental effects on the development of the fetus, it might be labeled prenatal child maltreatment.

## Discussion

### Limited knowledge of the antecedents of child maltreatment

The umbrella synthesis documents the evidence of intergenerational, psychological, physiological, and socioeconomic antecedents of child maltreatment. In our model of risk and protective factors (Figure 1), the intergenerational transmission of child maltreatment is one of the most robust effects emerging from our synthesis, with an effect size of nearly half a standard deviation. On the one hand, this illustrates a vicious cycle of maltreatment across generations as the effect size is rather large, certainly compared to the combined effect size for interventions focusing on prevention or reduction of child maltreatment. On the other hand, many parents maltreated in childhood escape this cycle of abuse. With a Cohen's *d* of .47, about 80% of parents with and without maltreatment experiences in their childhood show similar (non)abusive parental behavior, and there is only a 64% chance that a parent picked at random from the group with childhood maltreatment experiences will maltreat their own children than a person picked at random from the nonmaltreated group (probability of superiority, Magnusson, 2014). Consequently, maltreatment experiences are by no means doomed to be repeated in next generations.

The search for other antecedents of child maltreatment has still to get traction. We had expected to find more systematic reviews on parental psychopathology as a potential antecedent of child maltreatment, but no recent meta‐analytic syntheses emerged, pointing at a gap in the current meta‐analytical literature. However, some dimensions of personality within the nonclinical range such as insecure attachment styles or dependent personality were sufficiently frequently studied to allow for meta‐analytic synthesis, with a substantial overall effect size. Children of parents who experience intimate partner violence are also at increased risk of child abuse and neglect, compared with children in families without IPV. Intergenerational transmission of maltreatment might be (partially) mediated by personality issues such as insecure attachment styles or a dependent personality, and maltreatment experiences in childhood may have prepared individuals to approach conflicts in adulthood in a violent way, with their partner as well as with their children. However, only few studies examined mediation models such as proposed in Figure 1, and meta‐analytic evidence for mediation was absent. We do not know yet whether the mechanism explaining the influence of socioeconomic stressors or negative experiences of parents in their own childhood pertains to neurobiological dysregulation, or whether other mechanisms are at play.

As expected, low socioeconomic status was associated with increased risk of child maltreatment. It is tempting to suggest that screening for low education and unemployment would provide policy makers and practitioners with a target for effective (preventive) interventions. This may however be a problematic strategy for several reasons. Most importantly, this subgroup of families would only include a minority of the victims of child maltreatment; for example, in the Netherlands this minority would cover less than 10% of maltreated children (Euser et al., 2013). Early screening for low socioeconomic status might feed stigmatization and leaves the large majority of (potential) maltreatment cases out of sight, support and protection. In general, the four antecedents with robust meta‐analytic evidence (see Figure 1) each only account for <10% of the variance in child maltreatment, which leaves the largest part of the variance unexplained, even if the various antecedents would be unrelated. Added to this dearth of knowledge about antecedents of general maltreatment is the absence of replicable evidence on potentially divergent precursors of different types of maltreatment as specified in Figure 1. Comorbidity of maltreatment types might be one of the explanations for the absence of sufficient numbers of studies as input for meta‐analysis.

### Gaps in knowledge about neurobiological antecedents of child maltreatment

One of the goals of umbrella synthesis is to detect gaps in what we know about a research area such as child maltreatment. In our model of risk and protective factors, neurobiological antecedents have a prominent place (see Figure 1). However, robust empirical evidence on the neurobiology of maltreatment summarized in meta‐analyses is rather scarce. Most neurobiological studies on maltreatment have focused on the consequences of maltreatment experiences, for example, on brain structure and function (Riem et al., 2015). Meta‐analytic evidence on neurobiological antecedents is only available for higher baseline heart rate which was associated with increased risk for child abuse but with a small combined effect. Considering that low resting heart rate has been associated with antisocial behavior (Portnoy & Farrington, 2015), it is noteworthy that higher resting heart rate was found in maltreating parents and adults at risk for child abuse. The explanation may be that while antisocial behavior includes aggression and psychopathic behavior, which may be associated with callous/unemotional traits, callousness is not proposed to underlie child maltreatment, which may more often be the result of cumulative stress, including past trauma and socioeconomic difficulty. Unfortunately, no meta‐analytic evidence on parental neural or genetic antecedents of child maltreatment is available yet.

Nevertheless, it seems a plausible but potentially disturbing hypothesis that heritability might play a role in parenting (Euser et al., in press) and more specifically in intergenerational transmission of child maltreatment (Pittner et al., 2019). Heritability can be misunderstood to imply that children are to blame for their own maltreatment. However, it is the parents who are responsible for crossing the border of permissible parenting, because they are the more powerful party. Intergenerational transmission of maltreatment may occur due to heritable characteristics of parent and child but if parents were maltreated prevention efforts can be helpful, independently of the genetics behind transmission, parallel to the inherited condition of phenylketonuria (PKU) that can be cured by a change in diet. For example, in order to prevent or decrease the risk of child maltreatment, it might suffice to know that when children show oppositional behavior, some stressed parents are more prone to react aggressively while others tend to use sensitive discipline, regardless of the genetic underpinnings of the children’s or parents’ behavior. Either way, the focus of (preventive) interventions might be on alleviating (e.g., socioeconomic) stress and coaching the parents to use sensitive limit setting.

### Limited effects of interventions to prevent or curb child maltreatment

The umbrella synthesis shows the power of the antecedents of child maltreatment and at the same time the ‘power failure’ of the interventions to prevent or decrease child abuse and neglect in the next generation, with the effect sizes for intervention effectiveness being substantially smaller than the effect sizes for most of the antecedents of child maltreatment. An optimistic perspective is that home visiting programs might lead to a relative increase in CPS reports because interveners or nurses might be more alert to signs of maltreatment in the families involved in the intervention. But it may be more realistic to conclude that the scarcity of evidence‐based means to break the cycle of maltreatment is deplorable. Child maltreatment is a widespread, global phenomenon affecting the lives of millions of children all over the world, which is a betrayal of the United Nation’s Convention on the Rights of the Child (United Nations, 1995) in which the 194 ratifying countries (November 2009, with the notable exception of the USA, where the treaty is yet to be ratified) explicitly state that they will take all appropriate legislative, administrative, social, and educational measures, either nationally, bilaterally, or multilaterally, to protect children from maltreatment (Stoltenborgh et al., 2015).

The current types of home visiting and parenting programs seem insufficiently effective at significantly reducing the number of maltreatment cases reported to child protection services. This is partly due to the sample sizes common in this field. If we combine the effect sizes of the 11 meta‐analyses focusing on reduction of CPS cases (see Table 2 and Figure 3), the resulting effect size of *d* = .27 would require a sample of more than 700 families to reach statistical significance (alpha = .05, beta = .95; GPower 3.0). This would exceed the resources of most researchers or research teams. This effect size also points at the rather small practical results such interventions might be expected to have. As a thought experiment, with an estimate of Cohen’s *d* = .27, only 61% of the treatment group will be below the mean of the control group (Cohen's U3), 89% of the two groups will overlap, and there is a 58% chance that a person picked at random from the treatment group will have a lower chance to be reported to CPS than a person picked at random from the control group (probability of superiority). Moreover, 12 families would need treatment in order to have just one more favorable outcome in the treatment group compared with the control group. This means that if 100 families receive treatment, eight more will have a favorable outcome compared to those without the intervention, assuming that 20% of the control group have favorable outcomes, that is, improve compared to some predefined cutoff (Magnusson, https://rpsychologist.com/d3/cohend/). Of course, every child that is prevented from being maltreated is a precious accomplishment, but we should surely try and develop more effective interventions to protect more children and support more families.

A complementary preventive intervention approach that still has to be experimentally tested for its effect on child maltreatment is change in socioeconomic conditions of families in low‐resource environments. As we showed in the umbrella synthesis, limited social and material resources for families are among the most potent predictors and robust correlates of elevated maltreatment risks. More affordable education, universal (child) health care, more and better paid jobs, and paid parental leave may lead to less stress and better parental coping with challenging child behavior which in its turn may lead to lower basal physiological levels and lower risk of maltreatment (Reijman et al., 2016). In this respect, social and economic policies can make a substantial difference in the lives of overburdened parents and decrease the allostatic load interfering with sensitive interactions with their children. Allostatic load refers to the ‘wear and tear on the body’ due to chronic stress, having to cope with adverse events and environments (McEwen & Seeman, 2009). Large‐scale conditional or unconditional cash transfer experiments and experiments with vouchers to move to a lower‐poverty area might be utilized to demonstrate the efficacy of social changes for preventing or decreasing child maltreatment (Banerjee et al., 2015; Chetty, Hendren & Katz, 2016; Fiszbein & Schady, 2009).

### Combatting child maltreatment from a differential susceptibility perspective

The impasse of a vicious cycle of maltreatment and the failure to develop effective interventions might be broken in two ways. First, empowering parents to cope more sensitively with challenging child behavior and to set gentle but consistent limits to oppositional and coercive interactions is paramount in some of the more effective parenting interventions (e.g., the video‐feedback‐based intervention of Moss et al., 2011) which should be promoted and implemented on a wider scale. Importantly, the social context of such interventions should be taken into account. In a Dutch low SES sample, the nurse home visitation program proved to be effective in lowering the number of CPS registered maltreated cases in a preregistered randomized trial (Mejdoubi et al., 2015), but in the United Kingdom significant effects on maltreatment indicators seemed absent (Robling et al., 2016), which might point at the importance of contextual factors such as the level of care‐as‐usual in determining the success or failure of intervention programs (Olds, 2016).

**Figure 4:**
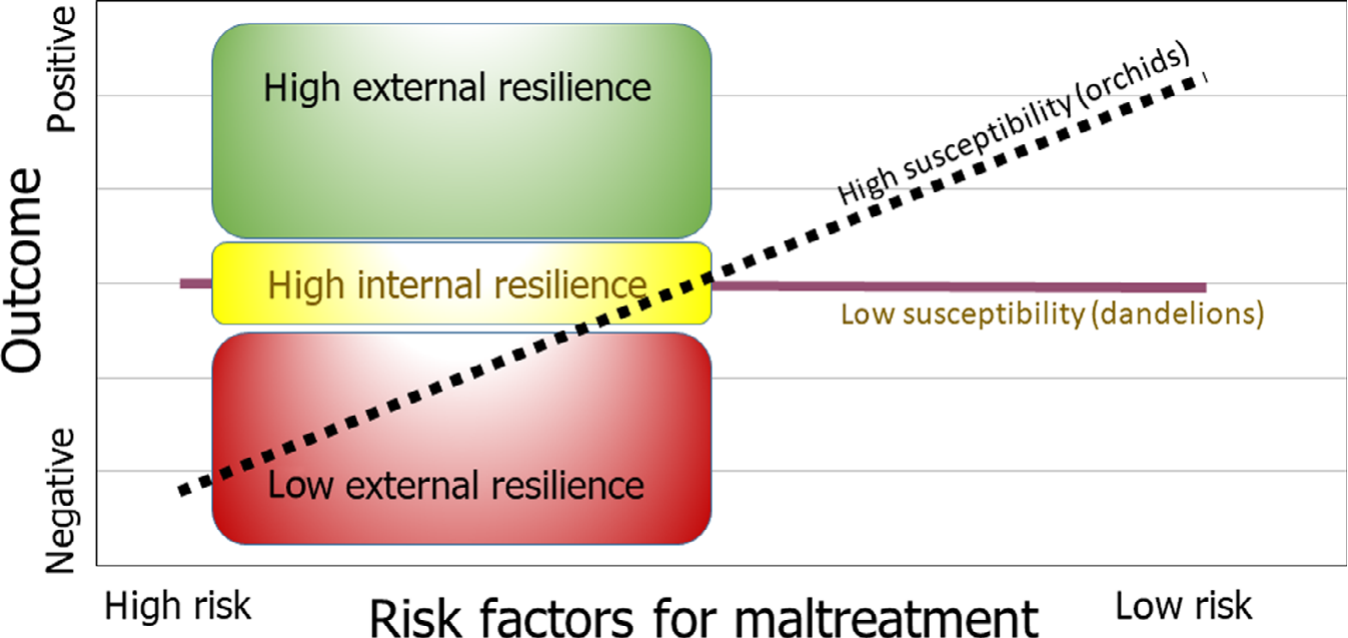
A differential susceptibility perspective on risk and resilience. Note: Resilient individuals show lower susceptibility to good as well as bad environments. More resilient, less susceptible parents might persist in ‘good‐enough’ parenting even in adverse circumstances, and more resilient, less susceptible children may survive maltreatment with fewer scars than their more susceptible but less resilient peers. The less susceptible individuals can rely on their constitutional or high internal resilience rooted in a less reactive temperament and neurobiological makeup, whereas the more susceptible individuals must rely more on a supportive environment as a buffer against adversities – which is high external resilience [Colour figure can be viewed at http://www.wileyonlinelibrary.com]

Second, we might address one of ‘the most provocative questions posed by … differential susceptibility’(Masten, 2019), namely whether the most vulnerable parents adapting poorly to adverse experiences might also profit most from changes for the better (see Figure 4). Differential susceptibility refers to the idea that some individuals are more open to environmental influences than others, for better *and* for worse (Belsky, Bakermans‐Kranenburg & van IJzendoorn, 2007). The failure to document intervention efficacy might not only be due to the inherent lack of effective components but also be caused by limiting the evaluation to average effects across all parents involved instead of looking for changes in the (a priori defined) most susceptible families: The efficacy of intervention effects on child maltreatment might be hidden in susceptible subgroups of participants (Bakermans‐Kranenburg & van IJzendoorn, 2015).

Differential susceptibility theory proposes three markers that differentiate the more susceptible from the less susceptible individuals, on the level of genetic, stress regulatory, and temperamental makeup (Bakermans‐Kranenburg & van IJzendoorn, 2015; Belsky & van IJzendoorn, 2017; Ellis, Boyce, Belsky, Bakermans‐Kranenburg & van IJzendoorn, 2011). Genetic markers have been found in genes involved in the dopaminergic and serotonergic system. In particular, the carriers of the dopamine receptor D4 7‐repeat alleles seem to be open to the environment, for better and for worse, as demonstrated in correlational as well as experimental studies (Bakermans‐Kranenburg & van IJzendoorn, 2015) and this genotype might be embedded in larger dopamine‐related genetic pathways or polygenetic susceptibility scores (Belsky & van IJzendoorn, 2017; Keers et al., 2016; Lemery‐Chalfant et al., 2018). Another marker of differential susceptibility is biological sensitivity to context, involving heightened stress and immune reactivity to negative stimuli in a chaotic and stressful environment, and at the same time elevated processing of positive stimuli in a structured, supportive environment (Boyce, 2019). Temperamental difficultness seems to be the most important temperamental marker of differential susceptibility, as documented in a recent meta‐analysis (Slagt, Dubas, Deković & van Aken, 2016). Difficultness is defined as a broad dimension encompassing (aspects of) negative emotionality, surgency, and effortful control, and it has been found to predict elevated levels of (internalizing and externalizing) behavior problems but also a heightened openness to the influence of a change in the environment for the better (Slagt et al., 2016).

In particular, difficult temperament and its adult personality corollary of orienting sensitivity (Rothbart, Ahadi, & Evans, 2000) or ‘sensory‐processing sensitivity’ (Aron, Aron, & Jagiellowicz, 2012) can be assessed rather easily and might be used to titrate intervention efforts and modalities to the degree of susceptibility of the parents and children at risk for maltreatment, and to evaluate interventions in a more focused way using *a priori* planned statistical interactions between experimental condition and these phenotypic markers of susceptibility (see Figure 1 for moderating protective factors). Genetic screening for differential susceptibility or assessment of biological sensitivity to context might be less feasible in practice and may certainly raise some thorny ethical issues. Even in the case of temperamental susceptibility, the ethical issue might be raised whether intervention efforts should be primarily directed at susceptible families. In our view, this question can only be answered affirmatively if sufficient basic universal support for all families is available (see also Bakermans‐Kranenburg & van IJzendoorn, 2015).

Through the lens of differential susceptibility theory, the question may be answered why child maltreatment is not always transmitted from one generation to the next, as documented in our umbrella synthesis. Masten (2001) defined resilience as ‘good outcomes in spite of serious threats to adaptation or development’. Resilient individuals are characterized by a lower degree of susceptibility to the environment. Less susceptible parents and children might show ‘good‐enough’ parenting in adverse circumstances or survive maltreatment with fewer scars than their more susceptible peers (see Figure 4). Less susceptible individuals can rely on their constitutional resilience rooted in a less reactive temperament and neurobiological makeup, whereas the more susceptible individuals must rely more on a supportive environment as a buffer against adversities (see the moderation by constitutional and contextual protective factors suggested in Figure 1). They may however flourish when they can grow up within a warm and sensitive caregiving scaffold. This role of social context in resilience has recently been highlighted by Masten (2019) who re‐defined resilience as the adaptive capacity of the developing system of relationships and social support in which the individual child is embedded.

It should be noted that differential susceptibility theory does not assume static markers of more or less openness to the environment. For example, set points for the stress regulatory system (e.g., functioning of the hypothalamic–pituitary–adrenal axis) may already be influenced by parental stress in the prenatal period (Boyce, 2019), and epigenetic changes of expression of structural DNA are partly under environmental control (Mulder, Rijlaarsdam, van IJzendoorn, 2017; Bakermans‐Kranenburg & van IJzendoorn, 2015) and thus dynamic. Even temperamental characteristics have been shown to be influenced by parental sensitivity in the first year of life (Hane & Fox, 2006). Thus, the three main markers of differential susceptibility should not be considered a lifelong fate but an opportunity for environmentally induced change.

Finally, we want to draw attention to the neglect of the prenatal period in theories and research on child maltreatment. Parenting starts as early as during pregnancy, and interactions between the fetus and both prospective mothers and fathers already begin to shape development after birth, for example through epigenetic changes (Mulder, Rijlaarsdam, van IJzendoorn, 2017; Glover, 2015). The prenatal start of parenting also signals the start of prenatal child maltreatment, arguably, for example, with continued smoking or alcohol consumption during pregnancy. Extreme alcohol intake might lead to fetal alcohol syndrome (Sokol, Delaney‐Black, & Nordstrom, 2003), whereas continued smoking may lead to changed methylation of growth factors such as IGF2DMR mediating the association of prenatal smoking with newborns being small for gestational weight (Bouwland‐Both et al., 2015). Fortunately, prenatal interventions of alcohol consumption of pregnant women (e.g., O'Connor & Whaley, 2007) were among the most effective interventions in the umbrella synthesis and deserve to be extended to other domains such as smoking and stimulating sensitive interactions between parents (Wang et al., 2017) and with the fetus (Bakermans‐Kranenburg, Juffer, & van IJzendoorn, 2018). Such prenatal parenting interventions might, together with social policies to alleviate the parental daily hassles and stressors, be crucial for preventing child maltreatment to emerge and to continue to damage children’s development.

## Supporting information


**Figure S1.** Coding form for meta‐analyses of maltreatment antecedents and interventions.
**Figure S2.** Effect sizes (d) for risk factors of child maltreatment in 2010 prevalence study in the Netherlands.
**Table S1.** Search terms and number of citations.
**Table S2.** Inclusion and exclusion criteria for the umbrella meta‐analyses review.
