# Supplementary material for: Annual Research Review: Umbrella synthesis of meta‐analyses on child maltreatment antecedents and interventions: differential susceptibility perspective on risk and resilience
Source: J Child Psychol Psychiatry. 2019 Oct 30;61(3):272–90. doi: 10.1111/jcpp.13147 (PMC7065145; doi:10.1111/jcpp.13147)
Supplement: Supplementary file 1 — Figure S1. Coding form for meta‐analyses of maltreatment antecedents and interventions. Figure S2. Effect sizes (d) for risk factors of child maltreatment in 2010 prevalence study in the Netherlands. Table S1. Search terms and number of citations. Table S2. Inclusion and exclusion criteria for the umbrella meta‐analyses review. [file JCPP-61-272-s001.docx]

**Supporting information – Umbrella synthesis of meta-analyses on child maltreatment antecedents and interventions: Differential susceptibility perspective on risk and resilience – by van IJzendoorn *et al*.**

**Figure S1.** Coding form for meta-analyses of maltreatment antecedents and interventions.

Author/title:

year:

coder:

Independent and dependent variable/ determinant and outcome:

1. Search strategy:

0) no systematic approach (3 different sources; three databases)

1) Systematic but not clear

2) systematic flow chart (e.g., PRISMA)

2a. N total number of subjects, if not exactly reported and cannot be computed: N< 1000 / N >1000

2b. k total number of study outcomes (if less than 4, do not include)

3. Study designs: Cross-sectional; longitudinal; RCT; quasi-experimental; mixed

4a. combined effect size

confidence interval

p-value

4b. *in case of trim-fill correction for publication bias:*

k trimmed studies

adapted effect size

confidence interval

p-value

4c. individual study effect sizes reported (in figure or table)? yes/no

if yes: N largest sample

effect size largest sample

4d. effect sizes for important moderators

5. Homogeneity test (Q or I-squared)

0) no homogeneity test

1) significant or large heterogeneity (sign Q or I >50%)

2) homogeneity (non-sign Q or I < 50%)

6. Publication bias

0) no publication bias test, or Not reported

1) publication bias, e.g. sign Eggers or sign correlation N – ES; skewed funnel; trim-fill needed

2) no publication bias, e.g. non-significant Eggers, funnel okay; no trim-fill needed

3) trim-fill applied, adapted effect size reported (see 4b)

7. Study quality ratings

0) no quality rating

1) quality rating done but not used quantitatively

2) quality rating correlated with ES

3) quality rating was not associated with ES

8. Intercoder agreement search

0) not reported

1) low (kappa <.50; % agreement < 70%)

2) satisfactory (kappa >. 50; % agreement >.70)

9. Intercoder agreement study characteristics

0) not reported

1) low (kappa <.50; % agreement < 70%)

2) satisfactory (kappa >. 50; % agreement >.70)

10. Intercoder agreement study quality rating

0) not reported

1) low (kappa <.50; % agreement < 70%)

2) satisfactory (kappa >. 50; % agreement >.70)

3) n/a, no quality rating

11. Intercoder agreement extraction statistics

0) not reported

1) low (kappa <.50; % agreement < 70%)

2) satisfactory (kappa >. 50; % agreement >.70)

**Figure S2.** Effect sizes (d) for risk factors of child maltreatment in 2010 prevalence study in the Netherlands.

Note: Based on sentinel reports, CPS reports, and self-reports (see Euser et al., 2013)

**Table S1.** Search terms and number of citations.

| # | Domain | # | Search Terms | Databases | | | |
| --- | --- | --- | --- | --- | --- | --- | --- |
|  |  |  |  | WOK | PubMed | Cochrane | |
|  |  |  |  | No of Citations (17.12.18) | No of Citations (17.12.18) | No of Citations  (17.12.18) | |
| 1 | Population | 1 | Child* | 1,062,877 | 390,046 | 1,407 | |
|  |  | 2 | Infant* |  |  |  |  |
|  |  | 3 | Adolesc* |  |  |  |  |
| 2 | Moderators/Outcomes | 4 | Maltreatment | 83,806 | 32,772 | 64 | |
|  |  | 5 | Abuse |  |  |  |  |
|  |  | 6 | Neglect |  |  |  |  |
|  |  | 7 | “Family Violence” |  |  |  |  |
| 3 | Review Design | 8 | Meta-analy* | 165,308 | 122,479 | 1,922 | |
|  |  | 9 | metaanaly* |  |  |  |  |
|  |  | 10 | “quantitative synthesis” |  |  |  |  |
|  |  | 11 | “research synthesis” |  |  |  |  |
|  |  | 12 | “quantitative review” |  |  |  |  |
|  |  | 13 | “systematic review” |  |  |  |  |
|  | Combined | #1 AND #2 AND #3  [01.01.2014 – 31.12.2018] | | 813 | 437 | | 19 |

**Table S2.** Inclusion and exclusion criteria for the umbrella meta-analyses review

| Domain | Inclusion | Exclusion |
| --- | --- | --- |
| Population | 1. Children and adolescents (i.e. under 18 years at time of maltreatment) | 1. Adult population (18 years or over at time of maltreatment) |
| Outcome/ moderators | 1. Reviews including papers on the impact/effects of maltreatment 2. Reviews considering maltreatment as an outcome variable. 3. Reviews that provide details about moderators of maltreatment. | 1. Maltreatment not considered 2. Unclear moderators of maltreatment 3. Not family violence |
| Design | 1. Quantitative reviews | 1. Review only narrative 2. No empirical data 3. No useable statistics |
|  |  |  |
